# Novel Mixing Relations for Determining the Effective Thermal Conductivity of Open-Cell Foams

**DOI:** 10.3390/ma15062168

**Published:** 2022-03-15

**Authors:** Jesus Nain Camacho Hernandez, Guido Link, Markus Schubert, Uwe Hampel

**Affiliations:** 1 Institute for Pulsed Power and Microwave Technology IHM, Karlsruhe Institute of Technology, Hermann-von-Helmholtz-Platz 1, 76344 Karlsruhe, Germany; guido.link@kit.edu; 2 Institute of Fluid Dynamics, Helmholtz-Zentrum Dresden-Rossendorf, Bautzner Landstr. 400, 01328 Dresden, Germany; m.schubert@hzdr.de (M.S.); u.hampel@hzdr.de (U.H.); 3 Chair of Imaging Techniques in Energy and Process Engineering, Technische Universität Dresden, 01062 Dresden, Germany

**Keywords:** open-cell foams, effective permittivity, thermal conductivity, platonic solids

## Abstract

This paper proposes a new approach to relate the effective thermal conductivity of open-cell solid foams to their porosity. It is based on a recently published approach for estimating the dielectric permittivity of isotropic porous media. A comprehensive assessment was performed comparing the proposed mixing relation with published experimental data for thermal conductivity and with numerical data from state-of-the-art relations. The mixing relation for the estimation of thermal conductivities based on dodecahedrons as building blocks shows good agreement with experimental data over a wide range of porosity.

## 1. Introduction

Effective permittivity, $\varepsilon_{\mathrm{eff}}$, and effective thermal conductivity, $k_{\mathrm{eff}}$, of open-cell foams are important properties for the design and optimization of microwave-heated elements [1]. Determining both effective properties for foams with a reliable mixing relation based on foam porosity (ratio of the void volume to the total foam volume), bulk properties and skeletal morphology would greatly facilitate the design of systems using microwave-heated elements. Such “cross-property” relations are commonly used to interrelate changes in the effective values of various physical properties (e.g., thermal conductivity, elastic moduli, electrical conductivity and fluid permeability) [2,3,4,5] caused by microstructural features (e.g., pores and inclusions) of heterogeneous materials. Consequently, estimating effective properties turns out to be more complex than calculating a weighted volumetric mean. Predictive relations for $\varepsilon_{\mathrm{eff}}$ or $k_{\mathrm{eff}}$ (or any cross-property) are based on models describing the microstructure of heterogeneous materials. Reliable predictions can only be obtained if the considered structural model resembles features of the foam microstructure. The geometrical representation of the foam morphology in the literature varies from the less accurate idealized assumptions (e.g., ordered, random and symmetrical distributions of solid and void phases) to more complex 2D structures (e.g., hexagonal honeycombs) and 3D unit cell morphology approaches (e.g., simple cubes, Weaire-Phelan unit cell and truncated tetrakaidecahedrons) [5,6,7,8,9,10,11,12].

Historically, the first relations formulated from idealized distributions of solid and void phases are based on the effective medium approximation (EMA) and belongs to the class of mean-field theories [5]. More recent relations are based on geometrical models considering details of the material structure, as well as structures modeled by using the finite element method (FEM).

In particular, for estimating the $k_{\mathrm{eff}}$ of open-cell foams, several relations can be found in the literature. These relations are mostly based on empirical data-fitting functions (e.g., Calmidi relation) [8], geometrical models (e.g., Bracconi, Dai and Yao relations) [9,10,11], probability distributions of parallel and series models (e.g., weighted arithmetic mean relation) [12,13,14], and those based on EMA assumptions, such as the Maxwell-type and self-consistent models (e.g., Differential Effective Medium relation) [12,15].

Recently, we proposed a numerical approach using FEM to derive predictive relations for the effective permittivity of open-cell foams based on two types of morphologies [16]. The first morphology corresponds to foams whose skeletons are based on Platonic solids (such as hexahedron, octahedron, icosahedron and dodecahedron) as building blocks, while the second morphology was reconstructed from micro-CT data of real open-cell ceramic foams. These relations based on Platonic solids and real open-cell foams are referred to as Platonic relation and OCF (open-cell foam) relation, respectively. Both relations agree well with the numerical data obtained from electromagnetic wave propagation calculations, as long as the foam behaves as an effective medium (effective medium approximation). In other words, on the macroscopic scale, foams behave similar to a homogenous medium. However, due to the lack of data, the relations are not yet validated at higher permittivity contrasts, where the EMA approach loses its applicability. Alternatively, the analogy of thermal and electrical networks can equally be used for the skeleton network of open-cell foams [9,17]. Here, extensive experimental and numerical data on the effective thermal conductivity of open-cell foams are available for validation. According to this analogy, it is reasonable to assume that any relation describing $\varepsilon_{\mathrm{eff}}$ can be used to estimate $k_{\mathrm{eff}}$, or vice versa as a cross-property relation [5,12,15,18]. Please note that only thermal conductivities are considered in this study and not convective heat-transfer coefficients or overall heat-transfer coefficients.

Following the thermal-electrical analogy, both Platonic and OCF relations can be used to estimate $k_{\mathrm{eff}}$. The Platonic relation for estimating $k_{\mathrm{eff}}$ corresponds to the following:

(1) $$k_{\mathrm{eff}}=\frac{-2P}{\left(1+P^{2}\right)}\left(k_{s}-k_{f}\right)+\left(k_{s}+gk_{f}\right).$$

where g is a correlation parameter that represents topological details of the skeleton morphology; P is the porosity; and $k_{s}$ and $k_{f}$ are the thermal conductivities of the bulk materials, i.e., the solid skeleton and the medium that fills the voids of the skeleton, respectively. For complex-valued quantities, such as $\varepsilon_{\mathrm{eff}}$ ($\varepsilon_{\mathrm{eff}}=\varepsilon_{\mathrm{eff}}^{\prime }-j\varepsilon_{\mathrm{eff}}^{\prime \prime }$), the calculation of g also gives a complex-valued quantity ($g=f\left(g_{m}^{\prime },-jg_{m}^{\prime \prime },g_{0}^{\prime }\prime -jg_{0}^{\prime \prime }\right)$) [16]. In contrast to $\varepsilon_{\mathrm{eff}}$, $k_{\mathrm{eff}}$ and, thus, also g are real-valued quantities ($g=f\left(g_{m}^{\prime },g_{0}^{\prime }\right)$). As a result, g is calculated as follows:

(2) $$g=g_{m}^{\prime }\left(k_{s}/k_{f}\right)+g_{0}^{\prime },$$

where $g_{m}^{\prime }$ and $g_{0}^{\prime }$ are expressed as follows:

(3) $$Y=\overset{6}{\underset{k=0}{\sum }}a_{k}P^{k},$$

with Y = {$g_{m}^{\prime }$,$g_{0}^{\prime }$}. The respective coefficients, $a_{k}$, are summarized in Table A1 of the Appendix A. Note that the Platonic geometry is no longer preserved if the size of the struts exceeds a certain limit and struts overlap each other, causing the closure of the open-cell faces. The porosity range in which foams exhibit the Platonic geometry is listed in Table A1. This corresponds to ideal Platonic skeletons and no to real foam skeletons where cell faces close at higher porosities (*P* ~ 0.5). The corresponding relation for estimating $k_{\mathrm{eff}}$ using the OCF relation is as follows:

(4) $$k_{\mathrm{eff}}=\frac{-2P}{\left(1+P^{2}\right)}\left(k_{s}-k_{f}\right)+k_{s}\left(1+P{\left(1-P\right)}^{3/2}\right).$$

The novelty of this work is the use mixing relations for $k_{\mathrm{eff}}$ predictions developed by using FEM electromagnetic wave propagation calculations to estimate the effective permittivity $\varepsilon_{\mathrm{eff}}$ of foams. Therefore, Equations (1) and (4) represent novel mixing relations for estimating $k_{\mathrm{eff}}$.

In the following assessment, predictions for the $k_{\mathrm{eff}}$ of open-cell foams using Platonic and OCF relations, as well as predictions from selected relations from the literature relations are analyzed and compared with experimental and numerical data.

## 2. Materials and Methods

The state-of-the-art $k_{\mathrm{eff}}$ relations [8,9,10,11,12,13,14,15] considered in this study are summarized in Table 1. They were selected because of their excellent prediction capability [8,9,10,11,12,13,14,15,19]. The considered skeleton materials of the open-cell foams and involved filling medium (as well as their bulk thermal conductivities, $k_{s}$ and $k_{f}$), and the open-cell foams data from which $k_{\mathrm{eff}}$ was obtained are summarized in Table 2 and Table 3, respectively [8,11,13,15,19,20,21,22,23,24,25,26,27,28].

For easier data comparison, the $k_{\mathrm{eff}}$ of open-cell foams is normalized as follows:

(5) $$k_{\mathrm{eff}}^{\prime }=\frac{k_{\mathrm{eff}}-k_{f}}{k_{s}-k_{f}}.$$

This way, all values are scaled between 0 to 1, allowing us to describe $k_{\mathrm{eff}}^{\prime }$ as a function of P, which depends on the foam microstructure and thermal conductivity contrast. The difference between $k_{\mathrm{eff}}$ estimated from relations and those from experimental or simulated data is quantified by the root-mean-square error (RMSE).

## 3. Results and Discussion

**Figure 1:**
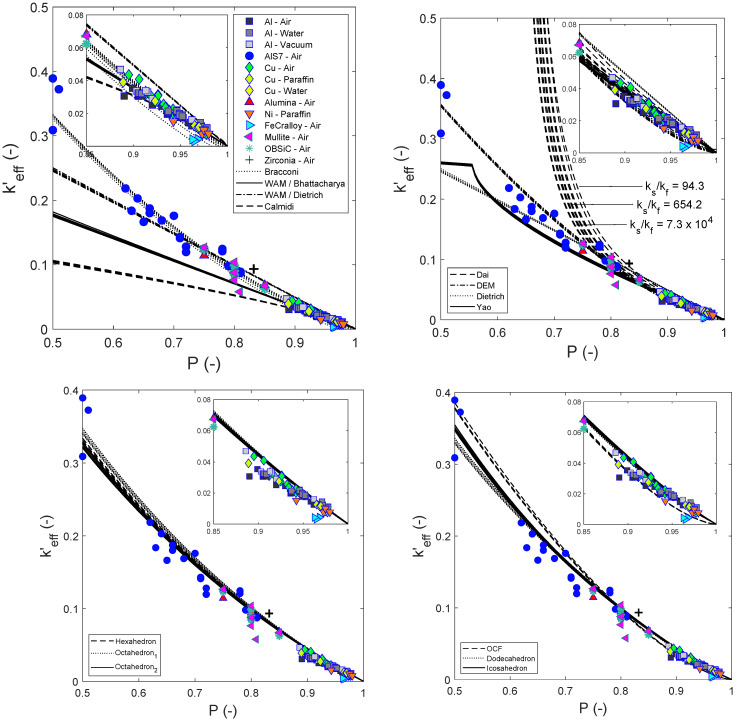
Normalized experimental ${k^{\prime }}_{\mathrm{eff}}$ values (represented by symbols corresponding to foams listed in Table 2) compared with those estimated from relations (represented by lines corresponding to relations listed in Table 1 and Equations (1) and (4)). The embedded subplots provide an enlarged view for the porosity ranging from 0.85 to 1.0.

Figure 1 shows the normalized $k_{\mathrm{eff}}^{\prime }$ data (symbols) found in the literature for open-cell foams against estimations (lines) using the literature mixing relations (upper figures), the Platonic relations (lower figures using Equation (1) based on different Platonic solids) and the OCF relation (lower-right subfigure using Equation (4)). As $k_{s}/k_{f}$ increases, $k_{\mathrm{eff}}^{\prime }$ decreases for both experimental data and estimates from relations (as illustrated in the right figure of the upper row for the prediction of the Dai relation [10]). In addition, Table 4 summarizes the RMSE of each relation at three different porosity ranges ([0.5, 1.0], [0.85, 1.0] and [0.9, 1.0]) to characterize the deviations from the experimental data.

Figure 1 (upper row) illustrates that the literature relations estimate $k_{\mathrm{eff}}^{\prime }$ better for P→1, and this is expected, because they were preferably developed for foams with higher porosity. The best predictions are obtained by the relation from Bracconi (see Table 4). However, it should be mentioned that the Bracconi relation accuracy decreases as $k_{f}$ increases, since the relation does not include a $k_{f}$ term.

The relations inspired by the Platonic solids (bottom row of Figure 1) provide excellent predictions with only minor deviations from the experimental data (see Table 4). The results suggest that the dodecahedral structure (followed by the icosahedral) best mimics the skeleton of real foams. It is important to highlight that other researchers [10,22] have proposed the Kelvin tetrakaidecahedron (known to reproduce packings with low surface area) as the best geometrical element to mimic foams. However, the modeling of this polyhedron has been performed only in two dimensions, using ligaments corresponding to struts and thus lacks important features, such as the effect of the geometrical shape of joints. Accordingly, a better description of tetrakaidecahedron-inspired foams would be required for a fair comparison with the dodecahedral and the icosahedral relations from this study.

The predictions from the OCF relation (see lower right graph of Figure 1) are similar to those of the DEM relation, which agrees with our previous study on the estimation of $\varepsilon_{\mathrm{eff}}$ [16] but deviates—to a certain extent—from the experimental data of $k_{\mathrm{eff}}$.

**Figure 2:**
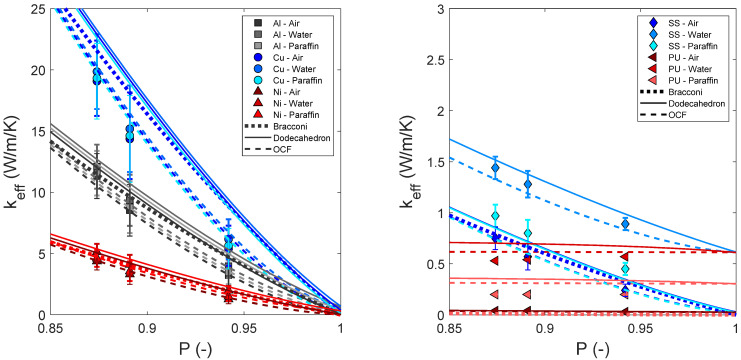
$k_{\mathrm{eff}}$ estimated from Bracconi, dodecahedron and OCF relations compared with those from numerical simulations of Al, Cu and Ni foams (**left**) and SS and PU foams (**right**). The errors bars correspond to the standard deviation as reported by August et al. [19]. Lines from Bracconi’s relation are overlapping, as it does not consider the filling medium.

Figure 2 shows the $k_{\mathrm{eff}}$ estimated from the relation of Bracconi [9] and from dodecahedron and OCF relations in comparison with those reported from numerical simulations [19].

Figure 2 reveals that, in contrast to the experimental values, the best predictions for the simulated data are obtained from the OCF relation (RMSE_OCF_ = 0.41 < RMSE_Bracconi_ = 1.27 < RMSE_Dodecahedron_ = 1.77). This is consistent with previous electromagnetic wave propagation calculations used for computing $\varepsilon_{\mathrm{eff}}$ [16] and with calculations via the diffuse interface representation of the phase-field model used for computing $k_{\mathrm{eff}}$ [19]. The structural models for computing $\varepsilon_{\mathrm{eff}}$ were reconstructed from tomographic scans of open-cell foams, while the structural models for computing $k_{\mathrm{eff}}$ correspond to synthetic foam structures using the algorithm proposed by August et al. [29]. Although the open-cell structural models used for the simulations are different, both are well represented by the OCF relation. Thus, it can be concluded that the simulation models are significantly different from real foams for the following reasons:

1. For the simulations performed to calculate $\varepsilon_{\mathrm{eff}}$, the skeleton morphology was reconstructed from µCT-scans of samples with porosities of 0.90 ± 0.01. At this porosity, both experiments and simulations are well estimated by the OCF relation (see Figure 1 and Figure 2). However, 3D erosion and dilation filters [16] were applied for generating models of different porosity, which—depending on the mesh resolution—may produce significant differences compared with the microstructure of real foams.
2. The synthetic foam structures generated by August et al. [19] are not morphologically identical despite having the same porosity. Numerically computed and measured experimental values of $k_{\mathrm{eff}}$ (as reported by August et al. [19]) only agree if the standard deviation is considered. This indicates that only a few of the synthetic structures are morphologically consistent with real foams.

## 4. Conclusions

Recently, relations have been derived to estimate the effective permittivity of open-cell foams based on two approaches: (1) using Platonic solids as building blocks of foam skeletons and (2) using the morphology of foam samples extracted from tomographic scans. Based on the thermal-electrical analogy, these relations can be used to estimate cross-properties, such as the effective thermal conductivity. In this work, an assessment of the predictions of the effective thermal conductivity of open-cell foams from these new relations has been presented. The relations have been compared with experimental and numerical data from the literature as well as with predictions from available mixing relations. It has been shown that foam’s thermal conductivity can be well estimated from the Platonic relation by using dodecahedrons (which describes foams based on dodecahedral building blocks).

The following recommendations can be derived from this work to properly select the most suitable relation:

1. For foams with porosities ranging from 0.5 to 1.0 and low bulk thermal conductivities of the filling medium, the Bracconi relation [9] is recommended.
2. Novel mixing relations are recommended for porosities ranging from 0.5 to 0.9, except for the Platonic relation based on dodecahedrons, which can be applied for porosities from 0.5 to 1.0. In addition, the Platonic relation based on dodecahedrons is recommended over the Bracconi relation for thermal conductivities of the filling medium higher than $k_{f}>1$ ${\mathrm{Wm}}^{-1}K^{-1}$.
3. The relations of Yao [11] and the weighted arithmetic mean approach with arithmetic coefficient proposed by Bhattacharya [14] are recommended if the porosity is higher than 0.85.

Finally, the simulated numerical data were well met by using the OCF relation. However, a significant difference was identified between the numerically predicted values and those from experiments.

## Figures and Tables

**Table 1:** Mixing relations applied for estimating the effective thermal conductivity of open-cell foams.

Relation	Expression	Remarks
Bracconi [9]	$k_{\mathrm{eff}}=k_{s}\left(\frac{2}{3}{\left(1-P\right)}^{2}+\frac{1}{3}\left(1-P\right)\right)$	$\mathrm{This}\;\mathrm{relation}\;\mathrm{ignores}\;k_{f}$ because it was derived from a correlation for the tortuosity in periodic ordered structures, which is defined by the skeleton structure only.
Weighted arithmetic mean (WAM) [12]	$k_{\mathrm{eff}}=\Psi_{\mathrm{arithm}}\left(Pk_{f}+\left(1-P\right)k_{s}\right)+\frac{1-\Psi_{\mathrm{arithm}}}{\left(\frac{P}{k_{f}}+\frac{1-P}{k_{s}}\right)}$	Weighted arithmetic mean of the Wiener bounds using the following: $\Psi_{\mathrm{arithm}}=0.35$ as proposed by Bhattacharya et al. [14] $\Psi_{\mathrm{arithm}}=0.49$ as proposed by Dietrich et al. [13]
Calmidi [8]	$k_{\mathrm{eff}}=k_{f}P+k_{s}0.181{\left(1-P\right)}^{0.763}$	
Dai [10]	$k_{\mathrm{eff}}=\frac{\sqrt{2}}{2\left(R_{A}+R_{B}+R_{C}+R_{D}\right)}\;$	$R_{A}=\frac{4d}{\left(2e^{2}+\pi d\left(1-e\right)\right)\left(k_{s}-k_{f}\right)+4k_{f}}$
		$R_{B}=\frac{\left(e-2d\right)}{e^{2}\left(k_{s}-k_{f}\right)+2k_{f}}$
		$R_{C}=\frac{2\left(\sqrt{2}-2e\right)}{\pi d^{2}\sqrt{2}\left(k_{s}-2k_{f}\right)+2k_{f}}$
		$R_{D}=\frac{2e}{e^{2}\left(k_{s}-k_{f}\right)+4k_{f}}$
		$d=\sqrt{\frac{\left(\sqrt{8}\left(1-P\right)-\frac{3e^{3}}{2}\right)}{\pi \left(3-e\left(\sqrt{32}+1\right)\right)}}$
		e=0.198
Differential Effective Medium (DEM) [15]	$\frac{k_{f}-k_{\mathrm{eff}}}{k_{f}-k_{s}}{\left(\frac{k_{s}}{k_{\mathrm{eff}}}\right)}^{1/3}=1-P$	Known also as the Bruggeman relation, non-symmetric.
Yao [11]	$k_{\mathrm{eff}}=\frac{1}{\left(\lambda /k_{E}+\left(1-2\lambda \right)/k_{F}+\lambda /k_{G}\right)}\;$	
		$k_{E}=\frac{\sqrt{2}}{6}\pi \lambda \left(3-4\lambda \right)\frac{1+b^{2}}{b^{2}}\left(k_{s}-k_{f}\right)+k_{f}$
		$k_{F}=\frac{\sqrt{2}}{6}\pi \lambda^{2}\frac{1+b^{2}}{b^{2}}\left(\frac{k_{s}}{2}-k_{f}\right)+k_{f}$
		$k_{G}=\frac{\sqrt{2}}{6}\pi \lambda^{2}\frac{1+b^{2}}{b^{2}}\left(k_{s}-k_{f}\right)+k_{f}$ λ is calculated (implicit method) from $P=1-\frac{\sqrt{2}}{2}\pi \lambda^{2}\left(3-5\lambda \right)\frac{1+b^{2}}{b^{2}},$ where a is a geometrical parameter (b=2.01 as recommended by Yao et al. [11]).

**Table 2:** Thermal conductivities of filling media and skeleton bulk materials (* average value [23]).

Skeleton Material	$\;\;\;\;\;k_{s}/Wm^{-1}K^{-1}$	Filling Medium	$\;\;\;\;\;k_{f}/Wm^{-1}K^{-1}$
Aluminum [8]	218	Air [11]	0.0265
Alumina [13]	25.9	Paraffin [11]	0.305
AlSi7 [15]	167	Vacuum * [23]	0.003
Cupper [11]	401	Water [11]	0.613
FeCr-alloy [28]	16		
Mullite [13]	4.4		
Nickel [25]	91.4		
OBSiC [13]	8.1		
Stainless steel (SS) [19]	15		
Zirconia [28]	2.5		
Polyurethane (PU) [19]	0.2		

**Table 3:** List of references with published $k_{\mathrm{eff}}$ values for different skeleton and filling media combinations and corresponding thermal conductivity contrast values.

Skeleton-Fluid	$k_{s}/k_{f}$	Skeleton-Fluid	$k_{s}/k_{f}$
Al-Air [8,19,20,21,22]	$8.2\times 10^{3}$	Nickel-Air [19]	$3.4\times 10^{3}$
Al-Water [8,19,22]	355.6	Nickel-Water [19]	149.1
Al-Paraffin [19]	714.7	Nickel-Paraffin [19,27]	299.7
Al-Vacuum [23,24]	$7.3\times 10^{4}$	Polyurethane-Air [19]	7.5
Alumina-Air [13]	977.4	Polyurethane-Water [19]	0.3
AlSi7-Air [15]	$6.3\times 10^{3}$	Polyurethane-Paraffin [19]	0.7
Cu-Air [11,19]	$1.5\times 10^{4}$	OBSiC-Air [13]	305.7
Cu-Paraffin [15,22,25]	$1.3\times 10^{3}$	Stainless steel-Air [19]	566.0
Cu-Water [11,19]	654.2	Stainless steel-Water [19]	24.5
FeCr-alloy-Air [26]	603.8	Stainless steel-Paraffin [19]	49.2
Mullite-Air [13,26]	166.0	Zirconia-Air [26]	94.3

**Table 4:** Comparison of the RMSE for $k_{\mathrm{eff}}^{\prime }$ of the considered relations (* weighted arithmetic mean approach with corresponding arithmetic coefficient proposed by Bhattacharya or Dietrich; see remarks in Table 1).

**Relation**	RMSE		
	P≥0.50	P≥0.85	P≥0.90
Bracconi	1.55	0.78	0.68
Calmidi	7.80	0.98	0.81
Dai	84.05	0.80	0.73
DEM	2.10	1.77	1.72
WAM/Bhattacharya *	5.38	0.68	0.58
WAM/Dietrich *	3.58	2.32	2.13
Yao	3.02	0.62	0.53
Hexahedron	1.85	1.29	1.14
Octahedron_1_	1.89	1.34	1.18
Octahedron_2_	1.90	1.32	1.18
Dodecahedron	1.65	0.87	0.73
Icosahedron	1.67	1.07	0.92
OCF	2.30	1.55	1.52

## Data Availability

Data are contained within the article.

## List of Abbreviations and Nomenclature

**Abbreviations**	**Description**
CT	Computed tomography
DEM	Differential effective medium
EMA	Effective medium approximation
FEM	Finite element method
OCF	Open-cell foam
PU	Polyurethane
RMSE	Root-mean-square error
WAM	Weighted arithmetic mean
SS	Stainless steel
**Nomenclature**	**Description (For dimensionless quantities, units are not indicated)**
a	Coefficients of the polynomials for calculating $g^{\prime }$
b	Geometrical parameter of Yao’s model [11]
ε	Relative permittivity
$\varepsilon^{\prime }$	Dielectric constant
$\varepsilon^{\prime \prime }$	Dielectric loss
d	Dimensionless foam ligament radius of Dai’s model [10]
e	Dimensionless cubic node edge length of Dai’s model [10]
	Thermal conductivity/${\mathrm{Wm}}^{-1}K^{-1}$
$k^{\prime }$	Dimensionless normalized thermal conductivity
Λ	Dimensionless parameter of Yao’s model [11]
	Dimensionless correlation parameter of the foam morphology
$g^{\prime }$	Real part of g
$g^{\prime \prime }$	Imaginary part of g
P	Porosity
R	Thermal resistance on a flux basis/$W^{-1}m^{2}K$ [10]
Ψ	Weight parameter [12]
**Subscripts**	**Description**
A, B, C, D	Unit cell subsection of Dai’s model [10]
E, F, G	Layer of unit cell of Yao’s model [11]
arithm	Arithmetic
eff	Effective
f	Filling medium
k	Degree of the polynomials for calculating $g^{\prime }$
m	Slope value of g
s	Skeleton
0	Ordinate-intercept value of g

## Appendix A

**Table A1:** Parameters used to calculate $g_{0}^{\prime }$ and $g_{m}^{\prime }$.

**Valid Porosity Range**	**Variable**	$a_{1}\;$	$a_{2}$	$a_{3}$	$a_{4}$	$a_{5}$	$a_{6}$
	Hexahedron						
0.058 to 1	$g_{m}^{\prime }$	0.5229	−0.9951	2.4460	−4.7673	3.9566	−1.1630
	$g_{0}^{\prime }$	0.2775	4.7271	−14.1449	15.0204	−6.6742	0.7939
	Octahedron_1_						
0.415 to 1	$g_{m}^{\prime }$	0.4008	0.6699	−3.2970	3.9169	−2.2435	0.5528
	$g_{0}^{\prime }$	2.1613	−7.0162	17.1386	−28.2326	23.5875	−7.6392
	Octahedron_2_						
0.093 to 1	$g_{m}^{\prime }$	0.6546	−2.1904	6.0160	−9.7020	7.2485	−2.0266
	$g_{0}^{\prime }$	0.3698	7.6377	−26.3063	34.1470	−20.6464	4.7987
	Dodecahedron						
0.078 to 1	$g_{m}^{\prime }$	0.4617	0.3546	−3.9082	7.6496	−7.0236	2.4661
	$g_{0}^{\prime }$	1.4799	−10.1720	50.1347	−109.2526	103.7717	−35.9641
	Icosahedron						
0.267 to 1	$g_{m}^{\prime }$	0.2213	4.1649	−17.8917	29.0420	−21.8497	6.3132
	$g_{0}^{\prime }$	2.4320	−21.2239	84.7916	−152.1911	124.4845	−38.2943

## References

[1] Vasudev H., Singh G., Bansal A., Vardhan S., Thakur L. (2019). Microwave heating and its applications in surface engineering: A review. Mater. Res. Express.

[2] Pabst W., Gregorová E. (2015). Critical Assessment 18: Elastic and thermal properties of porous materials—Rigorous bounds and cross-property relations. Mater. Sci. Technol..

[3] Schwartz L.M., Martys N., Bentz D.P., Garboczi E.J., Torquato S. (1993). Cross-property relations and permeability estimation in model porous media. Phys. Rev. E.

[4] Kachanov M., Sevostianov I., Barber J.R., Klarbring A. (2018). Chapter 6. Connections between Elastic and Conductive Properties of Heterogeneous Materials. Other Cross-Property Relations. Micromechanics of Materials, with Applications.

[5] Pietrak P., Wisniewski T.S. (2014). A review of models for effective thermal conductivity of composite materials. J. Power Technol..

[6] Krishnan S., Murthy J.Y., Garimella S.V. Direct simulation of transport in open-cell metal foams. Proceedings of the IMECE2005, 2005 ASME International Mechanical Engineering Congress and Exposition.

[7] Yang X.H., Bai J.X., Yan H.B., Kuang J.J., Lu T.J., Kim T. (2014). An analytical unit cell model for the effective thermal conductivity of high porosity open-cell metal foams. Transp. Porous Media.

[8] Calmidi V.V., Mahajan R.L. (1999). The effective thermal conductivity of high porosity fibrous metal foams. J. Heat Transfer.

[9] Bracconi M., Ambrosetti M., Maestri M., Groppi G., Tronconi E. (2018). A fundamental analysis of the influence of the geometrical properties on the effective thermal conductivity of open-cell foams. Chem. Eng. Process.-Process Intensif..

[10] Dai Z., Nawaz K., Park Y.G., Bock J., Jacobi A.M. (2010). Correcting and extending the Boomsma–Poulikakos effective thermal conductivity model for three-dimensional fluid-saturated metal foams. Int. Commun. Heat Mass Transf..

[11] Yao Y., Wu H., Liu Z. (2015). A new prediction model for the effective thermal conductivity of high porosity open-cell metal foams. Int. J. Therm. Sci..

[12] Pabst W., Hříbalová S. (2019). Describing the effective conductivity of two-phase and multiphase materials via weighted means of bounds and general power means. JOM.

[13] Dietrich B., Schell G., Bucharsky E.C., Oberacker R., Hoffmann M.J., Schabel W., Kind M., Martin H. (2010). Determination of the thermal properties of ceramic sponges. Int. J. Heat Mass Transf..

[14] Bhattacharya A., Calmidi V., Mahajan R.L. (2002). Thermophysical properties of high porosity metal foams. Int. J. Heat Mass Transf..

[15] Solórzano E., Reglero Ruiz J., Rodríguez-Pérez M., Lehmhus D., Wichmann M., De Saja J.A. (2008). An experimental study on the thermal conductivity of aluminium foams by using the transient plane source method. Int. J. Heat Mass Transf..

[16] Camacho Hernandez J.N., Link G., Schubert M., Hampel U. (2021). Modeling of the effective permittivity of open-cell ceramic foams inspired by platonic solids. Materials.

[17] Iasiello M., Savarese C., Damian P., Bianco N., Andreozzi A., Chiu W., Naso V. (2019). Modeling heat conduction in open-cell metal foams by means of the three-dimensional thermal fin theory. J. Phys. Conf. Ser..

[18] Sihvola A.H. (1999). Electromagnetic mixing formulas and applications. IEE Electromagnetic Waves Series: Institution of Electrical Engineers.

[19] August A., Reiter A., Kneer A., Selzer M., Nestler B. (2018). Effective thermal conductivity of composite materials based on open cell foams. Heat Mass Transf. Res. J..

[20] Phanikumar M.S., Mahajan R.L. (2002). Non-Darcy natural convection in high porosity metal foams. Int. J. Heat Mass Transf..

[21] Paek J.W., Kang B.H., Kim S.Y., Hyun J.M. (2000). Effective thermal conductivity and permeability of aluminum foam materials. Int. J. Thermophys..

[22] Boomsma K., Poulikakos D. (2001). On the effective thermal conductivity of a three-dimensionally structured fluid-saturated metal foam. Int. J. Heat Mass Transf..

[23] Schmierer E., Razani A. (2006). Self-Consistent open-celled metal foam model for thermal applications. J. Heat Transf..

[24] Sadeghi E., Hsieh S., Bahrami M. (2011). Thermal conductivity and contact resistance of metal foams. J. Phys. D Appl. Phys..

[25] Xiao X., Zhang P., Li M. (2014). Effective thermal conductivity of open-cell metal foams impregnated with pure paraffin for latent heat storage. Int. J. Therm. Sci..

[26] Coquard R., Rochais D., Baillis D. (2009). Experimental investigations of the coupled conductive and radiative heat transfer in metallic/ceramic foams. Int. J. Heat Mass Transf..

[27] Xiao X., Zhang P., Li M. (2013). Preparation and thermal characterization of paraffin/metal foam composite phase change material. Appl. Energy.

[28] Coquard R., Loretz M., Baillis D. (2008). Conductive heat transfer in metallic/ceramic open-cell foams. Adv. Eng. Mater..

[29] August A., Ettrich J., Rölle M., Schmid S., Berghoff M., Selzer M., Nestler B. (2015). Prediction of heat conduction in open-cell foams via the diffuse interface representation of the phase-field method. Int. J. Heat Mass Transf..

